# Psychological Quarterly Retrospect

**Published:** 1860-04-01

**Authors:** 


### *Psychological Quarterly Retrospect.*

Three questions of Moral Hygiene, of exceptional interest to the practical psychologist, have during the quarter been brought prominently before the attention of the public. We refer (1) to the probable influence of the Divorce Court upon the morality of the nation; (2), to the attempt made to introduce some knowledge of godliness among the nether classes of the Metropolis by holding religious services in the theatres; and (3), to the extraordinary efforts which have been made, also in London, in the form of midnight prayer-meetings, to work good among those unfortunate women whose calling has been characterized, by way of pre-eminence, as *the* "Social Evil."

Each of these questions may well be the subject of particular notice, and it is a notable fact that the two first-named have been submitted to grave discussion in our Houses of Parliament, than which, we think, nothing could more thoroughly testify to the soundness and vitality of the interest which the representatives of the nation take in its moral well-being.

The subject of the Divorce Court was brought before the House of Commons on the 7th of February, when Lord J. Manners moved for leave to bring in a Bill to enable the Court to sit with closed doors, and the remarks which he then made will best show the nature of the question involved in the discussion which followed. He said:—

"It was not his intention to re-open the discussions which preceded the establishment of the Court, or to say a word in connexion with the question how far it had an effect upon the public morality and domestic purity of the country. The evil for which he wished to provide a remedy was purely accidental, and quite unnecessary as a concomitant to the new law. Great stress had been laid upon the advantage of avoiding the indecent details which used occasionally to transpire in actions for *crim. con.* and in divorce cases before the House of Lords. But, he asked, had any change for the better really taken place? The reverse was the case, and the question had now come to this, that a man must either discontinue taking in the daily papers during the sitting of the Divorce Court, or he must consent to place in the hands of his family details of the most indecent and abominable character. It was the saying of an heathen poet of corrupted Rome, '*Maxima debetur pueris reverentia,*' and, acting on this maxim, the Legislature two or three years ago, on the suggestion of the present Lord Chancellor, passed a law to put a stop to the sale of indecent publications. But the evil which had resulted from the indecent publications of Holywell-street was far less extensive than that caused by the compulsory publication of the proceedings in the Divorce Court, because when a man went into a shop to purchase one of those publications it was with his eyes open—he knew perfectly well what it was he was going to get; but, in the other case, wherever the English language was read, details equally corrupt and disagreeable were by a sort of unfortunate fatality spread broadcast. Hence it was that the feeling had been produced in the public mind to which he now asked the House to pay respectful deference. The Attorney-General in the discussion last year painted in strong, but not too strong, language the evils which would result from compelling the Court to sit with open doors. He pointed out how a young, innocent, and pure-minded woman might be compelled to give evidence of the most excruciating nature in that Court, crowded as it notoriously was with the most abandoned of both sexes, before the recording pens of the representatives of the public press, and suggested whether, rather than submit to such a terrible ordeal, many women might not shrink from asking for that relief and justice to which they were entitled. He left it to those who presided over and practised in this Court to say whether that anticipation had been realized. He was informed that it had been, and if that were so, to the argument of decency and morality he added this further motive of doing justice to unfortunate individuals. It was not his intention to quote any cases from the proceedings of the Court. He rested his case entirely on the notoriety of the facts, and if they were not thoroughly known to all the members of the House, he would not ask them to legislate. The remedy which he proposed to apply was that suggested by the able judge who presided over the Court, and sanctioned by the highest authorities in the House of Lords and the law officers in the Lower House. It was simply to give the Court the power of sitting with closed doors whenever it was deemed expedient—a power which was possessed by the Ecclesiastical Court and the House of Lords when between them they managed the divorce business—a power possessed by the Court of Chancery and by the analogous Courts in Scotland, France, and other countries, and which by universal testimony might be safely entrusted to the eminent judges who presided in that Court. He was at a loss to see how his proposal could be so objectionable as to procure for it the unusual honour of being opposed at that stage. He might be told that it was an old adage, 'Publicity is the soul of justice,' to which he would reply by another adage, 'No rule without an exception,' and the exception in this case was in the paramount interest of decency and morality. Nine-tenths of our statute law were exceptions from some general rule. The House was waiting with great anxiety the speedy restoration of the Chancellor of the Exchequer to health, in order to hear from him what had induced the Government to depart from the well-known received rule of political economy by negotiating a commercial treaty with the Emperor of the French. On the notice-paper for to-night there were no less than three notices, which were in direct violation of some received principle of political economy, and the Act to which he had already referred, for putting down the sale of indecent publications, was in direct violation of the principle—'An Englishman's house is his castle.' Perhaps it might be said that the remedy was unnecessary, since it was already in the power of the Court to close the doors; but whatever might have been the case previously, that argument could no longer be used after the introduction of the Bill of last year, which showed that the presiding judge, the legal authorities of the House of Lords, and the law officers were of opinion that he did not possess the power of closing the doors of the Court until the Legislature gave it by statute. No one, he thought, would contend that this power might not be exercised as advantageously for the public interests by the judges of this Court as it had heretofore been exercised in the Courts of Chancery; and if such a discretion were permitted, to whom could its exercise more properly be entrusted than to that distinguished man who presided over the Court of Probate and Divorce? In bringing forward this Bill he was actuated by no indirect wish either to damage or to prop up the new system of divorce by law. He scorned the motives which had been imputed to him, and he attributed no unworthy objects to others. The evil, he contended, was fatal, flagrant, and increasing; the remedy was simple, obvious, and innocuous; and it came before them recommended by the heads of the law, and by the very founders and framers of that Court in which he proposed that it should be put into operation. If there were those who believed that any other remedy was capable of being applied to this admitted evil, let them propose their plan, and he would most cheerfully support it; but, meanwhile, he entreated the House not to withhold its sanction from the introduction of a measure directed to such a desirable end, and recommended by so great a weight of authority."

It was unfortunate that several of Lord J. Manners's observations were open to the satirical remarks which Mr. Roebuck immediately attached to them. But the speech of this last-named gentleman throws too great a light upon the whole subject not to be quoted in full. He said:—

"A remark had once been made by Swift, which the present motion of the noble lord fully verified. This was, that 'the nicest persons had the nastiest ideas.' The proposition of the noble lord he believed to have originated in the same state of feeling that possessed the American lady whose sentiments of modesty were wonderfully offended by the naked legs of her pianoforte. If the noble lord had taken into account the end which was sought to be attained by the Divorce Court, he would, he thought, have arrived at a different conclusion. That Court had been created for a mixed object: it affected not merely private rights and obligations, but also public obligations. It not only determined whether a man should be divorced from his wife, but it went further, and declared why he should be divorced, and by that means it affected public morality. If the Court was shut up, one of these great means of influencing the public would be utterly taken away. He was aware that the English notion of morality was confined to picking a pocket and seducing another man's wife. But there were other things which he read daily in the papers by which he was much more affected. He constantly saw in the reports of the Police Courts that men were brought up for threshing their wives, almost beating out their eyes and nearly taking away their lives. These things went on from day to day, and disgraced the people of England in the eyes of Europe and of the world. Another great object which had been effected by the publicity of proceedings in the Divorce Court was the dispelling of the idea that immoral practices were confined to the upper and lowest classes of society; and it had been shown that among the middle class what was particularly recognised by Englishmen as immorality was as rife as in any other, and he thought this a very great benefit; he hated shams, and he believed the morality of the middle class to be as great a sham as had ever existed. There ought, then, to be a degree of healthy hardihood in a man's character which would enable him to bear this exposure. The noble lord had stated that it was impossible to put a newspaper into the hands of one's wife or daughter. This statement he denied altogether; and he maintained that nothing had been more creditable than the conduct of the public press on this question. They had the most filthy details before them—ay, and from the middle class too, and they had not shocked any decent man's or woman's mind by what they had disclosed. But let the truth be told; was it right that the middle class should continue to appear as models of angelic purity, when they were as earthly and immoral as any other class? The noble lord stated that the judges of the criminal courts had the power of shutting out women and children. Yes; but how was it done? In a criminal case, the judge has the depositions before him, and knows what is going to be proved is of a character that women and children had better not hear. The Judge of the Divorce Court could not do this; and as a judge would act only on his individual caprice, he would ask the House whether it could expect from a court of justice the advantages it was intended to produce, if it was left to the caprice of the judge to decide what was fit to be made public. Each judge would act on his own opinion: one might exclude the public, the next might take a different view of the case. Then they might have a prurient judge—there were such things—who might delight to make jokes before his Court that he would not dare to utter if the press was present. Even now, with the control of the press, he could put his finger on judges whose jokes did no honour to an English court of justice. One great use of a court of justice is to serve as a guide to the morality of the people. If the proceedings were secret, they would seem to be a thousand times worse than anything the public was likely to read. The motion of the noble lord, therefore, though made with the best possible intentions—hell was paved with them—would do the greatest possible mischief, and he should oppose it."

Little can be added of weight to these observations of Mr. Roebuck's, but we would insist on the importance of the view which he so clearly set forth, that the published proceedings of the Divorce Court were a great gain to the kingdom, because they made us aware of a huge moral evil festering among the middle classes, of which it may be said we had before but imperfect ideas. Our readers will perhaps remember that we had previously adopted this view of the question in assigning (with all due respect be it said) to Sir Cresswell Cresswell the part of Asmodeus, and that we had pointed out his court as one in which could be seen unveiled some of the innermost workings of the moral life of our society.[1] Those who had not been startled into a conviction, by the huge trade frauds which have of late been so common among us, that no small amount of the seemingly high moral polish of the middle classes was but a veneer of sanctimonious theory,[2] can scarcely resist the conclusion that such is the case, as it is forced home to them by the revelations of our Divorce Court. It is disclosures like these which so effectually aid in scraping off the whitewash with which we have bedecked our civilized sepulchres, and so doing enables us more effectually to bring to bear upon the moral evils which work among us that healthy appetite for doing good, which we fully believe to exist in no small degree in this land, and of which the very discussion in question is a happy instance, although it may have gone a little astray.

This, however, does not touch the core of Lord J. Manners's objections. For it would avail little if, while gathering good on the one hand, we should be scattering evil broad-cast on the other. Now he classes the reports of trial in the Divorce Court, which appear in the daily papers, and the indecent literature of Holywell-street together, as being of an equally injurious character as affecting morality;

[1] See *Retrospect*, January, 1860.
[2] "Veneered by sanctimonious theory."—*Tennyson*.

but the reports of the Divorce Court have a more extended influence for evil on account of the more widely-spread diffusion of the medium through which they are made public. We are entirely at issue with Lord J. Manners on the essential point of this opinion.

We have, from our literary duties, to keep a tolerably sharp eye upon the reports of divorce proceedings, and in no single instance can we call to mind any details made public which could be supposed to exercise an injurious effect upon the morality of an individual, *unless that individual's morals had been previously tainted.* There must have existed, first, a capacity for and a sensitiveness to appreciate the filthy facts hinted at, rarely, if ever, specified in the reports of cases. It may be true, as Lord J. Manners states, that the Divorce Court is a favourite haunt of prurient individuals; but this assertion has no weight as indicative of a probability that the reports of the courts are effective as *initial* causes of immorality. We should have supposed that any pure-minded male or female would rather learn another lesson from the perusal of these records—a lesson taught by the terrible destruction of domestic happiness, and of all the holy ties of domestic life there exhibited—a lesson, to wit, of which the first manifestation is a sound-minded terror of immorality, the second, a strengthened resolve to avoid even the faintest shadow of it.

Again: we know nothing personally of the Holywell-street literature referred to, but we were so astonished at its being classed by Lord J. Manners as of a like character with the reports of the Divorce Court, and we entertained such very different views of it, that we have taken the trouble to make some inquiries respecting it. It was merely necessary to listen to the titles of the chief books coming under this denomination, to ascertain how greatly Lord J. Manners had erred in comparing them with the reports of the Divorce Court. The indecent literature of Holywell-street *has for its sole object to create and foster licentiousness, and it is specifically written and published for this end.*

It is not to the transactions of our law courts that we must look for active agents in spreading and aiding immorality. We must look somewhat deeper. The efficient causes of vice do not usually thrust themselves boldly upon the surface of society. Alluding to the Holywell-street literature recals to our mind that a short time ago, while in one of the chief libraries in the metropolis—one as much frequented by ladies as gentlemen—we espied upon the table a new French work, of somewhat tempting promise as a book for a lazy hour. The title was *Romans Parisiens*, the first and chief one being headed *La Vertu de Rosine.* The story was written with charming crispness and elegance; but the subject was simply the apotheosis of an example of illicit but ungratified love, which was wound up by suicide of the most artistic description. Books of this class are by no means tabooed, it is to be feared, in many English houses and English libraries; and the unswerving object of these works is to throw a guise of respectability and *virtue* (as the tale we have just referred to) and right over those very immoralities which choke our Divorce Court with cases; these books, in short, are neither more nor less than the *recherché* varieties of the indecent literature for which Holywell-street is so infamously renowned.

Lord J. Manners's Bill was rejected, and the more general reasons which justified this rejection are thus expressed by the *Times* (February 6th), in an article written the day preceding the introduction of the Bill:—

"Lord John Manners proposes to introduce a Bill to-morrow giving the Judges of the Divorce Court the power of directing that any particular cause shall be heard with closed doors. Such a measure will, no doubt, attract the sympathies of a great number of people. It is at first sight an effort in favour of morality and purity, and it is easy enough to stigmatize those who oppose it as partisans of indecency and epicures in scandalous details. But it will become the House of Commons to decide the question on reasonable and constitutional grounds, unmoved either by prejudices or sneers. That there is a good deal to be said on both sides we freely admit; but, on the whole, we are in favour of upholding the decision of the House last year, and making no change in the law.

"The arguments of those who would confer an exceptional power on the Judges of the Divorce Court will, of course, be based on the recent narratives of domestic unhappiness which have been given to the world. But we may be allowed to say that the gentlemen who would make these cases the ground for an amendment of the law are mostly like Lord John Manners himself, persons of impulse rather than judgment, and entirely unacquainted with the working of our tribunals. He has, we feel sure, no clear notion of the scope of his own proposition, and those disposed to vote with him are only actuated by a feeling that the trials in the Divorce Court contain very improper details, and that it is very wrong in the newspapers to publish them. It is for the House, however, to look deeper, and to decide the question like statesmen and jurists. The actual state of things may be soon described. It has always been thought necessary that trials in this country should be conducted openly, with free access to as many of the public as the court will hold. That publicity is the best safeguard against unfairness has passed into a truism. So continually has this doctrine been acted upon, that in our criminal courts matters of the most revolting kind are inquired into publicly. Details worse than anything which can possibly be brought before the Divorce Court are given in evidence at the Old Bailey or the Assizes, and no power of excluding the public has ever been assumed by the judges. A girl must come forward in the presence of one or two hundred men to testify to her own violation, and to stand the cross-examination of the prisoner's counsel. Other matters of a grosser kind, which are unreported in newspapers, also occupy the Court, in the presence of a throng of loungers. All that the judges assert their right of doing is to direct that women and boys shall leave the court. This being the state of things with regard to all the Courts in the kingdom, the question is whether there is anything in the causes tried in the Divorce Court which calls for an exceptional power. We think there is not. Undoubtedly much that passes there is unfit for the public eye, but that is a question between the press and the public, and not to be solved by any clumsy legislation about sitting with closed doors.

"When the Court was first established the Judges did certainly on one or two occasions exclude the public. But when these cases are examined they furnish the most conclusive argument against Lord John Manners's Bill. The subject is not an attractive one, but still in a matter like this it is necessary to throw aside false delicacy, and point out facts which amateur legislators forget. It may be said, then, that the causes which the Court ordered to be heard privately were not mere histories of matrimonial infidelity or quarrelling. There is a class of cases of so melancholy a nature, and involving details so harrowing and humiliating, that it has been thought but a proper concession to human feelings to screen them from the public ear. Such cases have been heard in private, and could they be always distinguished from others where the passions or perversity of the parties are the cause of the suit, no one would refuse to give the Judges a discretion. But it will be seen at once that narratives which have moved Lord John Manners to introduce his Bill have nothing to do with the class of cases in which the Judges have asserted the right to close the doors of the court. People have been shocked by the disputes of the Allens, the Rowleys, and others; but, supposing Sir C. Creswell to have the power of hearing causes in private, does any one think that the learned Judge would have excluded the public on any of these trials? There is nothing to distinguish them generically from the every-day causes brought before the Court. A counsel cannot move to have a petition heard privately on the ground that the evidence is likely to be interesting or laughable, or that the lady or the gentleman is known in London society, or that the letters are written in a style of absurd sentimentality. And yet it is these things which make people read the trial! If more harm is done by one story than by another, it is only that the one is, if we may call it so, more picturesque than the other. Here, then, lies the weakness of Lord John Manners's proposition. He would give the Judges a discretion in excluding the public. But it is certain the Judges must either hear every cause in private, and so make the Court a perfectly secret tribunal, or they must continue to hear publicly the very kind of causes which are the occasion of the present scandal. The Judges would never consider it consistent with their duty to hear any matters in private, except those to which we formerly alluded. Now, as these are always omitted from newspaper reports, the public would gain nothing by the change in the law. The usual run of divorce trials would be heard with open doors, as before, and the details would be spread abroad just as they have been within the last few months. Lord John Manners's Bill must therefore either be nugatory, or authorise a system of secret trial, which the country would not submit to for a month.

"We cannot think that the House will assent to the measure of Lord John Manners, but there is no doubt that the feelings which have induced him to bring it forward are shared by a large number of well-meaning persons. The Court of Divorce, by cheapening a process which formerly could only be resorted to by the rich, has roused a number of persons throughout the country to seek for relief from the burdens of an unhappy marriage. The stories are no worse than those which used to be told in the Ecclesiastical Court, in trials for criminal conversation, and before Committees of the House of Lords; the only difference is that there are more of them, and that, the Divorce Court being new, and having been discussed with great earnestness by two parties in the country, the reports of the trials are eagerly read. Our advice is to leave things alone, and trust to the good sense and moral feeling of society. Undoubtedly, the newspapers have a great responsibility in this matter, and any one of them pandering to the grosser tastes of its readers will deserve and receive public reprobation. But it must be remembered that the task of the press is no easy one. To omit all notice of the proceedings of the Court would be to encourage the very evil which sensible men most fear—namely, collusion. Where a couple could go quietly into court and get divorced without the world knowing anything about it, the Judges would indeed have need of all their vigilance. Reported, then, the cases must be, and the only question is whether the report should be a brief or a full one. Whatever be its length, it must, however, present a fair view of the case. In justice to the judges and the jury a newspaper has no right to omit important matter on the ground of its indelicacy, when the omission makes the verdict or the judgment seem grossly unjust. The public justly takes upon itself to review the decisions of our Courts of Law, and, accordingly, it must not object that the newspaper reports on which its opinions are to be grounded sometimes contain what is unpleasing to a delicate mind. But this is a question to be settled by time and good sense; the press will be bound by its own interest to conform to the laws of decency, and then the presence of a few loungers in Sir C. Cresswell's court will be a matter of little importance."

Several most important hints on the effect of legislation as an agent expressive of certain forms of immorality may be obtained from the recently published French criminal statistics. The points to which we refer have been so ably set forth in a leading article of the *Daily Telegraph* (March 16th), that we shall content ourselves with quoting this, merely observing, that we are not to be understood as assenting to all the opinions which the writer expresses:—

"A well-known English judge once boldly asserted that the cause of every crime upon which he had to adjudicate could be traced directly or indirectly to the prevalence of intemperance. Unfortunately for the acceptance of this rash dictum, and within a few months of its delivery, a perfect epidemic of financial crime set in among the educated classes. Staid and pious bankers pawned their customers' securities; barristers and solicitors absconded; dilettante transfer clerks and philanthropist railway officials committed gigantic forgeries; and trusted servants made off with boxes of gold-dust. It was evident that the tavern or the beershop could have been little instrumental in inducing the commission of these misdeeds. A year after this the Indian rebellion broke out, and the "mild Hindoos" who never ate flesh, and never drank ardent spirits, plunged into a saturnalia of outrage, plunder, and slaughter. The judge's proposition was *primâ facie* so evidently untenable, that it were scarcely worth while to combat his fallacies, or even to notice them, save in so far as to express a regret that he had not been discriminating enough to point out the fact which must be acknowledged by all thinking men: that certain classes of crimes in England, notably assaults on women, poaching, onslaughts on the police, and ill-usage of children, were mainly caused by habits of intoxication. Crimes against life and against morality may be always, without dogmatism, ascribed to the ineffaceable existence of a principle of evil in the human heart. Crimes against property, petty larceny, and vagrancy, are almost entirely attributable to the want of schools and to the paucity of soap and water. When the very humblest members of the community are taught to read and write, and to wash themselves thoroughly, no English judge will go circuit without being presented in at least one assize town with a pair of white gloves.

"We yesterday published, in our Paris correspondence, some very interesting statistics relative to crime in France, which we gathered from the official report of the administration of criminal justice in 1858, and were then compared with former years. Were there anything valid in the irrational reference of *all* criminality to intoxication, the French criminal calendar should be almost a *tabula rasa*. Our neighbours are even better off than teetotallers, for they have self-command enough to be cheerfully abstemious, to preserve a highly cultivated taste for generous wines and good cognac, but to indulge in every description of fermented liquor only in strict moderation. As the French are, however, great consumers of tobacco, it might be assumed, *cæteris paribus*, that the innumerable cigars and pipesful of *caporal* they puff in the faces of the passers-by may have something to do with their offences against the law; indeed, we have Mrs. Partington's dictum that 'smoking, where there's curtains, is next to manslaughter.' At all events it cannot be denied that a drunken man in France is next to a monstrosity, and that, as a nation, the French are eminently sober in their habits. Unfortunately, when we come to examine the statistics we have mentioned, we find that, with all their moderation in the use of cakes and ale, the liege subjects of the Emperor Napoleon are by no means so virtuous as might be expected. It is true that the aggregate of accusations against individuals has decreased since 1854, when it amounted to 5525, to 4302 in 1858. The decrease seems generally to have been in petty thefts, mendicity, and vagabondage. But, on the other hand, there is a marked and ghastly increase in serious offences. Assassination has risen from 184 to 196; murder—we think that the crime of *meurtre*, answering to our manslaughter, is hereby meant—from 99 to 114; parricide—a crime almost unknown in England—from 12 to 17; wounding followed by death from 61 to 82; other serious wounding from 54 to 65; and violence in families from 50 to 57. Thus, while we find a diminution in those comparatively trifling delicts brought on by poverty and destitution, a frightful aggravation has taken place in crimes springing from the worst passions of humanity. The criminal has now a full belly, and no longer steals; but, in lieu of purloining a loaf or picking a pocket, he sharpens a knife and stabs his neighbour. A still larger proportion of increase is visible in another and repulsive class of crimes. There were 238 cases of outrage upon women in 1858, against 188 in 1854. There were 784 cases of criminal assaults on *children*, against 617. As regards both women and children, the outrages are understood to mean that which in England is known as the capital offence.

"While coining, uttering base money, arson, and miscellaneous crimes have decreased, perjury, subornation, and fraudulent bankruptcy have risen in number. Infanticide has regularly increased since 1851, when the total of *discovered* cases was 164. It is now 224. In fact, crimes of immorality seem to have made gigantic strides since Louis Napoleon, with a tardy puritanism, gave greater powers to priests and confessors; enjoined the police to enforce with greater rigour the disciplinary control over prostitutes; forbade the most trivial pamphlet or almanack to be sold without the police seal of colportage, lest it should contain matter dangerous to religion or morality, and compelled the *étalagistes* of the Quays and the Palais Royal to pull down the naughty lithographs that garnished their walls. Would that his Imperial Majesty could put a stop to the importation into this country of the abominable French photographs and stereoscopic slides which, glued up in the pockets of portfolios, or forming the false bottoms of trunks, still continue to baffle the vigilance of the officers of the English Customs! There is matter also for grave reflection in the fact that in the country where these hideous outrages on women and children take place, the publication of reports of certain trials in courts of justice is invariably prohibited; that the ravisher is arraigned *à huis clos*, in the strictest privacy, and that in domestic life the youth of both sexes are kept apart with almost conventual strictness. Nor should it be forgotten that a *bureau de mœurs* forms an important section of the prefecture of police; that public women are not allowed to be seen on the Boulevards, in the gardens of the Tuileries, or in the Champs Elysées; that they cannot exercise their sad vocation without a regular licence and permit, revocable at the pleasure of the police, and that after eleven o'clock at night they are confined, under heavy penalties, to their habitations.

"The perceptible increase of criminality in France cannot be chargeable to the severity of the criminal code. When its provisions are legally administered, that code is the most lenient in Europe. Anterior to the first revolution, it was comparatively Draconic, and the gibbet, the stake, the wheel, the pillory, the scourge, and the branding-iron alternately plied their functions on the Place de Grêve. Torture was a recognised part of the judicial system. Far different doctrines now prevail. The guillotine is the *ultima ratio*, but its ministrations are resorted to with the extremest rarity. 'Extenuating circumstances' are admitted in cases of deliberate murder, and many a wretch who in England would swing gets off in France with a term of hard labour in the penal settlements. The average punishment for commercial forgery (*faux en ecritures prioc*é) is five years at Toulon, often only *réclusion* in a *maison centrale*. Corporal punishments, save on refractory convicts, are unknown to the French law. Only the worst of criminals are fettered. And yet with this mild and equable code crime has increased and continues to increase in France. May it not be somewhat feasible that, in a country where every canon of public morality has been successively overthrown, and where the memory of a great political crime, begun in perjury, fructified by bloodshed, culminating in despotism, is yet fresh in the memories of the population, faith in and respect for the dictates of morality in private life should have sustained a heavy blow, should have fallen into contempt and neglect? A people who have no means of expressing their opinions, who are not allowed to listen to the counsels of those qualified to instruct them; who are not permitted to meet, to write, to speak together in freedom, become, for all their 'material prosperity,' arrant slaves, and are apt to acquire servile vices. When the Roman people, in the decadence of their empire, were most enslaved and most civilized, they were most corrupt and most depraved. Freedom flourishes best in a pure soil."

We may add, as a note to this article, that our contemporary's conceit of the self-command of our neighbours, and the generous wines they consume, is very pretty, and may, to some extent, apply to the educated classes ; but in other respects it is a formidable error. Count de Montalembert, addressing the National Assembly, said in 1850 that "Where there is a *wine-shop*, there are the elements of disease, and the frightful source of all that is at enmity with the interests of the workman ;" and Quetelet states in his work *Sur l'Homme*, that "of 1129 murders committed in France during the space of four years, 446 have been in consequence of quarrels and contentions in taverns."

The establishment of religious worship in many of our metropolitan theatres is certainly one of the most extraordinary manifestations of the philanthropical activity of the day. At the first glance there is something so repulsive in the step that we are apt to pay slight heed to the motives which have led to it. Yet these are such, as set forth in Lord Shaftesbury's most noble speech upon the subject in the House of Lords (February 24th), that we must perforce admit a justification of the movement. It is undoubted that there is a vast mass of the London population who are relatively in darker heathenism than even the Fejee islanders were in their primitive state; it is certain that the means at command for carrying civilization into this mass were entirely insufficient, if not wholly unfitted; and there can be no doubt that the present novel method had recourse to, may have, if it has not already had, the effect of breaking up the harsh, unyielding soil, prolific of little else than crime, and thus exposing it to higher fertilizing influences. It was not a question of what Lord Shaftesbury and his coadjutors would like to have done, but what they could do, under what they rightly regarded as an imperative necessity, which had been too long left in abeyance. Of course, there are many who would have left the sheep struggling in the ditch until the presumed orthodox engineering apparatus had arrived, by which the animal could be extricated; but we would fain believe that the animal and its Proprietor will be more gratified when, thinking solely of the good, some one or other jumps at once into the ditch, and raises or attempts to raise the fallen creature once more to the enamelled surface of the meadows.

It may be, moreover, that these services will react on the theatres themselves, and so, at one and the same time, elevate the character and standing of the people to whom we are indebted for a chief amusement, and the people who constitute one of the most powerful causes of the moral deterioration of a nation. We have had occasion before to insist upon the conclusion, that the vices of each class of society act upon every other class, however remote—a truth which, if it were appreciated more sensitively, would perhaps excite a more earnest attention among the mass of the people to the moral renovation of the nether-classes. We cannot detach ourselves from the evil influences which emanate from the uttermost sections of the population; we are inextricably linked to them by our trade and domestic relations.

The raid against the *Social Evil* existing in the metropolis is even more remarkable than the religious services in the theatres, and it constitutes so novel an infraction of the routine usually adopted in dealing with the subject that we shall not hesitate to devote a little space to the matter. The question involved is one of the vexed ones of moral therapeutics, and hence of high interest to the practical psychologist.

The new method which has been had recourse to for the purpose of effecting some good among the unhappy "street-walkers," consists in the institution of midnight prayer-meetings in the immediate vicinity of their chief haunt. The nature of the first meeting (the type of those which have succeeded) is thus narrated in the *Times* :—

"One of the most extraordinary meetings for years past took place yesterday morning (or rather the proceedings were to have commenced at midnight, on Tuesday), at the St. James's Restaurant, St. James's Hall, 69, Regent-street, in connexion with the important question of the great social evil.

"The meeting was none other than one of 'fallen women,' for the purposes of hearing prayer and addresses, and originated in this manner:—Some gentlemen connected with the Country Towns' Missions, English Monthly Tract Society, Female Aid Society, London Female Preventive and Reformatory Institution, the Trinity House, and other institutions, feeling anxious for the welfare of the multitude of fallen women who congregate every night in the Haymarket, Regent-street, and the principal casinos and cafés in the neighbourhood, resolved, after mature consideration, to attempt to convene some of those unhappy persons in a suitable place near those localities, where judicious addresses might be given, to be followed by prayer. The mode which to the conveners appeared most likely to succeed was to invite them to tea and coffee; and a neat card, enclosed in an envelope, was distributed among them at the casinos, cafés, and in the streets, indited as follows:—'The favour of your company is requested by several friends, who will meet at the St. James's Restaurant, 69, Regent-street, to take tea and coffee together, on Wednesday night, February 8, at 12 o'clock precisely.' Some hundreds of these were distributed during the past few days, and, as will be seen, the experiment met with a great amount of success, notwithstanding that many treated the matter with ridicule, while numerous others thought the whole affair a hoax. Such, however, it did not turn out to be; for shortly before midnight a large number of these unfortunate creatures arrived at the entrance of the St. James's Restaurant. Here they were shown into the large dining room of the hall, capable of holding some hundreds of persons. There was an abundant supply of tea and coffee, with bread and butter, toast, and cake, to which the strange assembly did good justice, at the various tables about the room, and round which they clustered in small parties of six or eight, chattering over the peculiarity of the meeting, amd wondering what was to be the course of proceeding. The number gradually increased till there must have been at least 250 persons present, and these were solely composed of the unfortunate creatures whose moral and social condition the meeting had been convened to ameliorate, excepting some thirty or thirty-five clergymen and gentlemen who had been instrumental in calling the meeting. Of course, the meeting was not allowed to be a public one so far as regarded the admission of the other sex; for, had it been so, no doubt a very large number would have assembled out of curiosity, if not for any worse motive. While the repast was going on the principal gentlemen present mustered together at a conspicuous spot for the purpose of addresses being delivered to the meeting. Among them were the Hon. and Rev. Baptist Noel, the Rev. W. Brock, the Rev. W. O'Neill, the Rev. — Haughton, Mr. Latouche (the banker), Mr. W. J. Maxwell, Mr. Theophilus Smith, and others.

"Shortly after one o'clock the Rev. W. Brock stepped forward and briefly opened the proceedings by stating the object of the meeting.

"The Hon. and Rev. Baptist Noel then addressed those assembled in an eloquent, yet pathetic and affectionate discourse, alluding to his hearers as his 'dear young friends.' He commenced by drawing a picture of the history of a virtuous woman from her childhood, pointing out the unspeakable love of the father and mother for the child, the association with sisters and brothers, the affection of the husband, and at last the love which she herself bears her own children; and then he compared that picture with the position of those who had erred from the paths of virtue. It was quite possible, however, he assured his hearers, that some of them might yet be happy; they might ask him how, and say it was difficult to become so—and so it was, he admitted, but it was not impossible, for they had a friend who was even more tender than the mother, and stronger in his love than the father; and One who would never desert them. He was a friend who would rescue them if they trusted in His boundless confidence. That friend was Jesus their Saviour, who had died for them; He was with them in that room, as certain as possible, and just ready to be their friend; therefore he entreated them to turn to their Saviour. Their whole future depended upon whether they would have Him or not; He could take them to glory from a life which must end in perdition, could cleanse them of their sins, and carry them to God. If they asked him when to do it he should say at once, and they would be happy for the rest of their lives; if they believed in Him they would be saved. The Saviour himself said, 'God so loved the world that He gave His only begotten Son, that whosoever believeth in Him should not perish, but have everlasting life.' Would they accept the offer or not? He entreated them to accept it at once and be happy for ever, their cheeks never fading, their conscience would sleep in peace, and they would live long to enjoy the esteem of those who were good. Let them take the resolution at once, and they would never regret it. The hon. and rev. gentleman then read letters from several girls who had been reclaimed, stating the happiness they felt, and then he went on to say that his young friends might ask how could they follow the course he pointed out. Of course, it would require some sacrifice to be made, but they must expect that, and help each other, and it would not be a matter of regret ultimately that they had made the sacrifice. They might think they would never be loved again, but he told them they would. Therefore, let them say like others, "Let us make the sacrifice;" for was it not better to be happy for ever than to walk on to the end which was perdition? In conclusion, he exhorted them not to depart without heeding what he had said. Might the Lord accept his prayers, and might He also accept those unfortunate young creatures he was addressing, and to them he said, 'Give up that which is contrary to the will of Jesus and say, "I will take heart and be a child of God."'

"The Rev. W. Brock, Rev. Mr. Haughton, Rev. W. O'Neill, and others then offered up prayers, and the effect produced by the earnest and touching appeal of the first-named gentleman, delivered in a deep tone of voice, was most touching.

"It was announced that any present who repented their sins would be received into the London Reformatory or the Trinity Home, and that further arrangements would be made for the reception of others elsewhere if funds could be provided.

"The meeting broke up about three o'clock.

"The conduct of those present was highly creditable, and quite void of levity or contumely, and we may safely say that the experiment so far has been successful."

The following comments of the *Daily Telegraph* (Feb. 11) may be regarded as pretty well exhausting the stock objections to this movement:—

"Nothing could be more attractive to benevolent minds than a rational project for reclaiming and restoring to society those poor fallen women who crowd our streets at night. Once show how this object may be effected, and the entire community would be carried away by impulses of sympathy. But is the great social evil to be obliterated by midnight meetings of unfortunates, by cards distributed in casinos and other places, by tea and coffee, bread and butter, toast and cake? We give the gentlemen who assembled on Thursday morning at St. James's Restaurant, St. James's Hall, credit for the best intentions, but, at the same time, we think they understand nothing whatever of the world. What was the purpose of their strange convention? To pray with the outcasts, to speak of redemption to sinners, to reform the wicked. They set about this laudable effort in a most singular manner. They invited the wanton sisterhood to an entertainment combining the spirit of a conventicle with the attractions of an evening party. There were to be speeches and refreshments, touching appeals and buttered toast, offers of pardon and muffins. Who can say how many went to the meeting as to a tea party, who thought of it seriously, and who carried away its reminiscence as a jest? We remember what happened when Mr. Henry Mayhew, prompted by kindly feelings, summoned the ticket-of-leave men to be lectured. They came, heard, dispersed, and no one ever knew of one black sheep in that grotesque congregation as having been bleached into a sense of propriety. So, we fear, will it be with the pitiable frail ones who met at St. James's Hall early on Thursday morning. About two hundred and fifty of them were gathered together; they were entirely in the dark as to the meaning of the summons which had been addressed to them. A neat card, enclosed in an envelope, had been circulated, setting forth that 'The favour of your company is requested by several friends who will meet in the St. James's Restaurant, 69, Regent-street, on Wednesday night, Feb. 8, at twelve o'clock precisely.' What could this polite invitation imply? Perhaps an extraordinary revel; not impossibly—the tea and coffee being introduced to veil the dramatic surprise—a champagne supper. Might it not be a regular carnival? At all events the guests mustered, wondering how the mystery might be revealed, and numbers of them, no doubt, were disappointed when they saw Mr. William Brock, Mr. Baptist Noel, and Mr. Theophilus Smith doing the honours. Thither had they come, says the report, very fashionably dressed, in a blaze of trinkets and jewels, the tinsel gewgaws that conceal the dust and ashes of their lives; and what was the festival? Tea, coffee, bread, butter, toast, cake, and sermons, to the substantial elements of which they rendered 'ample justice.'

"But it must not be supposed that the preaching was thrown away so far as the emotions of that particular evening were concerned. Many of the unhappy creatures buried their faces in their handkerchiefs and sobbed aloud. More than one had to be removed from the room in an unconscious condition. The spiritual drug, following upon the carnal refreshments, had been adroitly administered. But was it legitimate to make the experiment, and can we hope that one jot or tittle of good will be effected by it? Here was an invitation anonymously distributed in the streets and casinos, inviting the prostitutes of London to a sort of conversazione; they congregated long after midnight, decked out in all their gauds; they partook of the refreshments provided, while 'wondering what was to be the course of proceeding.' Wondering, indeed! What else could they do at that unseemly hour, in that conspicuous room, two hundred and fifty of them face to face, mutually acknowledging and thereby aggravating their degradation, with the streams of London vice and infamy flowing past the door? At three o'clock in the morning, excited and bewildered, they were let loose upon the pavement in throngs which must have astonished the late wayfarer. Do the reverend gentlemen who got up this eccentric demonstration believe that one of these poor things is less a harlot now than she was on Wednesday evening? What religious or moral purpose did Mr. Noel serve when he drew the portrait of a woman virtuous from her cradle? The creatures present fell not within that category. Many of them wept, no doubt; some fainted: but all returned straight into the world of paint, and lace, and silk, of glitter, immodesty, and excitement; and we may be sure that it will be long before the assemblage will be forgotten as a burlesque and a mockery. Not that we deem these unhappy wanderers irreclaimable; not that we rebuke a Christian preacher for calling them his 'dear young friends.' Would that public philanthropy exerted itself even more persistently than now to redeem them from the perdition of their shameful careers! Their pretended gaiety is wretchedness; their laughter is cynicism; their finery conceals the skeleton of the pauper. More than most evil-doers are they to be pitied, and even at the worst shall it not be tolerable to pardon and bring back within the human fold a weak one whose errors Heaven, more merciful than the world, would forgive? Therefore it is not because we thus characterise the effort to reclaim a certain proportion of these girls that we deprecate such exhibitions as those of Thursday morning. Receive them, by all means, into the London Reformatory or the Trinity House; but refrain from melodramatic appeals, from midnight celebrations in the neighbourhood of the Haymarket, from mysterious proposals of tea and coffee, which bring together two hundred and fifty prostitutes, proclaiming their humiliation in a multitude 'most fashionably attired, and displaying large quantities of jewellery.' Two or three who remember their homes and families may sob, and some may swoon upon the recollection of their guilt; but the gin palace is at hand, the throng will burst upon the streets, the hour passes, the figure of the preacher vanishes, his voice is no longer audible; some profane Pompadour or Dubarry of the *pavé* insinuates a jeer, and instantly the whole effect of the exhortation is dissipated. It is very likely that, as is stated, there was no levity or contumely so long as the proceedings lasted; but does any one imagine that the dawn of Thursday saw one of Mr. Noel's penitents stripping off the tawdry badges of her disgrace, and resolving henceforth to lead a life of morality and indigence?

"We will anticipate the answer in the sense most favourable to the philanthropists of St. James's Hall. Perhaps two or three girls have applied to the reformatories; they have, we assume, been admitted; and they will, we hope, be reformed. But might not a far better result ensue from the working of benevolent schemes more judicious and less obtrusive? There is a smack of quackery about the Regent-street congress which we find it difficult to pass without observation. It is very fine to talk of snatching brands from the burning, of clearing casinos and depopulating coffee-houses; it sounds highly creditable to give the fallen a last chance; but charity and religion have found other and better means of doing their work. The noiseless steps of the apostle, the unostentatious voice of the cottage or attic visitor, the melting pleading of the quiet missionary, have told upon the consciences of the wicked, the desperate, and the abandoned; but what benefit ever arose from philanthropic tea-meetings, at which hundreds of sinners, after being duly petted with earthly refreshments, were adjured to reflect upon the responsibilities of their souls? The spectacle reminds us of those Jesuit propagandists in the East, who invariably commenced their Catholic ceremonies by bowing down to an idol. They first conciliated the native Pagans and then denounced idolatry. Thus, Mr. Brock and Mr. Noel laid a strong foundation of bread and butter before they ventured to speak of chastity, and sugared the coffee half an hour in advance of the perorations which threw several congenial subjects of their spiritualism into ephemeral convulsions. We repeat that their enthusiasm was probably meritorious; but, like gunpowder, benevolence is a very destructive element in the hands of those who misunderstand its application."

We shall not discuss the objections advanced by our contemporary. The movement has been carried into effect, and many "unfortunates" have been persuaded to attempt to save themselves. This is so far well; but we think that the remote results of the movement will in all probability be more truly important than the immediate.

An immense and hitherto insuperable obstacle to working any permanent good in the *Social Evil* has been the determined opposition of the bulk of the people to acquiring any information about it, and consequently a very general neglect of it. Those who listen with rapt attention to tales of revolting immorality and vice among heathen nations, when told from the "Foreign Missionary platform," or recounted in Foreign Missionary works, and who, so appealed to, will open their purses as wide as their ears, are too apt not only to close both the one and the other when appealed to on account of the *Social Evil*, but are even too much disposed to regard in an unfavourable light those who venture to offend their ears with the subject. Pity it is that there are so many good people who, to use Uranie's happy expression, in *La Critique de l'Ecole des Femmes*, "Etoient plus chastes des oreilles que de tout le reste du corps."

Now the midnight-meetings, and the character of the gentlemen and ladies who have conducted them, will, we hope, have the good effect of breaking down, in no small degree, the injurious barrier of mistaken modesty which has driven the subject of the *Social Evil* into the shade. In this way a re-action on the male sinners may be brought about, by inducing a less heedless tone of feeling among society towards the active agent in the unhappy woman's fall. On the other hand, the greater interest excited may induce greater attention and greater pecuniary liberality towards the subject, and thus make feasible the establishment in greater number of the more permanent agencies for the relief of the evil; such agencies as we possess in our admirable town-missionaries, and as we ought to possess in *matrons*, who would devote themselves to a mission of peace and love to their erring sisters,—their efforts being rightly supported by the establishment of temporary or permanent refuges where needful.

While the three questions that we have just considered stand most prominent among the matters of the quarter which more immediately concern us, there have not been wanting other events which serve to mark the steady progress of that active, right-minded philanthropy of which these questions are so marked an illustration. We may refer, for example, to the Bill which has recently passed for limiting the hours of labour of women and children in bleaching and dyeing works, and which will doubtless exercise as beneficial an influence on the moral and physical condition of the females and young persons engaged in those occupations, as was effected by the Short-Time Bill affecting the same classes of individuals working in woollen manufactures. *Apropos* of the first-named Bill, the *Times* (March 23, 1860) has well remarked that—

"The people of England have solemnly proclaimed that the principle of *Laissez-faire* shall not apply to the using up of human flesh and blood. That women and children are not free agents, and that society in its corporate capacity has a right to interfere for their protection, is a principle which it is now too late to combat. Indeed, the Legislature has gone much further, as in the acts for the regulation of shipping, where seamen and emigrants are placed under the special protection of Government, and the doctrine of non-interference in private contracts utterly repudiated. Far above the advantage to society of material gain is the advantage of a vigorous and healthy people, and if a cancerous growth of dwarfishness and imbecility is allowed to come into existence in any part of the island, no one can tell what may be the consequences in the next age. In spite of all the care of Parliament and all the labours of sensible and right-minded employers, the population of a Lancashire town cannot be contemplated without a certain misgiving. Watch them as they pour along the streets to dinner, observe their pale faces, their stoop, their thin hands, and their somewhat unsteady gait, and it will glance across your mind that the first place in the commerce of the world may be too dearly purchased. But, whatever they may be, it is the universal testimony of the Factory Inspectors that the evil would have been far worse without Government interference. Mr. Horner, in his report for the half-year ending the 31st of October, 1859, says, 'The experience of twenty-six years convinces me that the legislative interference for the regulation of the labour of children, young persons, and women, in factories, is now viewed by the great majority of the occupiers as having done, and as continuing to do, a great amount of good without any interference with the prosperity of their trade.' Mr. Horner is sanguine respecting the employment of children. He thinks that under the present law they are not overworked, that the three hours they get in school daily give them the rudiments of education, while their employment sharpens their faculties and gives them independence at an early age. Mr. Baker says, 'I think I can show that the Factory Acts have put an end to the premature decrepitude of the former long-hour workers; that they have enlarged their social and intellectual privileges; that by making them masters of their own time they have given them a moral energy which is directing them to the eventual possession of political power, and that they have lifted them up high in the scale of rational beings, compared with that which they had attained in 1833.' So much for the right of Parliament to interfere with the labour in factories, and for the consequences which this interference has produced.

"Turn now to the Bleaching-works. Nothing in the annals of Manchester or Stockport, in the days when Lord Ashley carried on his campaign against the mill-owners, can be compared with the revelations which have been lately made respecting the condition of these abodes of misery. Mr. Roebuck only quoted a few of a whole series of heart-rending narratives. We may give some further extracts. One youth says, 'At times, if a sudden order has come, we have not been in bed more than sixteen or eighteen hours a-week.' Another says, 'At my commencement' (when he was a child of eleven) 'I began at twelve o'clock on Sunday night, and worked till eight o'clock on Monday night. Then we started at six o'clock on Tuesday morning, and worked till twelve at night; and we did this for two or three months without stopping.' Wright Mather says, 'I am foreman of the clamping-room. In summer I have seen the room at 130 degs.; in winter it is generally about 80 to 100 degs. We have come at six a.m. and worked till nine p.m. all last week. . . . There are four clamping-rooms, and when they are all a-gate there are sixteen females at work in them. In the other drying-place there are five boys; they work very often sixteen, seventeen, and eighteen hours a-day. The heat is very great in that room—from 100 to 150 degrees, and that is under the mark.' James Thomson, of Mr. Wallace's works, Burnbank, Glasgow, says, 'We employ about ten men and forty females; in summer, some of the hands work occasionally from six a.m. to twelve p.m. the whole week through; we did this last summer several times. Three days we worked twenty hours each day. The *ages of most of our girls are from ten to eighteen*. . . I feel when I am urging the females to work these long hours that I am doing what is not right, but I have been urged to do it to get a lot of goods finished. Sometimes they stay here all night, and then we make a place for them to lie down upon in a store-room, upon the pieces of goods unfinished. Sometimes fourteen or more girls will pass the night in this manner after working nineteen hours, and coming out of those hot places dripping wet with perspiration, and their clothes wet through with it.' We feel that we need add nothing to this horrible picture. If such revelations do not rouse society to rescue these girls and these children from their living death, then do we as a nation deserve the worst that the world can say of us, and an hour of just punishment will inevitably arrive."

Conspicuous among the events of the quarter have been the recurrence of several most brutal outrages on board American ships in British waters. Events of this class are sufficiently common in our own mercantile navy to justify an inquiry into the causes which lead to their repetition, and some portion of the active philanthropical spirit which we have seen conspicuous in the subjects that have already occupied our attention, might, perhaps, with advantage be devoted to such an inquiry. It would seem, however, as if outrages of the character referred to were far more common in American ships than our own. This fact is of considerable interest in connexion with others which appear to point to a growing *fashion* for deeds of violence among an influential section of the Americans. The sources of this peculiar tendency would form a curious and instructive psychological study, for which, however, we do not possess the materials. Such facts as do from time to time come to the knowledge of the English reader are not without considerable interest as bearing upon this question. The account and comment of the *Times* (January 17, 1860) upon one of the most serious recent examples of barbarity in the American marine, may be quoted with advantage:—

"There must be some hidden cause acting on the American people and producing in them a certain savageness of temper, which, increasing year by year, threatens to become the most marked feature of their character. Their forefathers in the first days of the Republic do not seem to have possessed it, nor had it a few years ago risen to the height which it has now attained. Ferocious duels and assassinations at home and deeds of still more revolting violence on helpless subordinates at sea are ever being recounted of Americans who seem to be taken as a fair specimen of their countrymen, and, however accounted for, there seems, unhappily, little doubt of the fact that a people sprung mainly from the same stock as ourselves are becoming singularly addicted to violence and cruelty.

"No more horrible story has ever been told, even of the American mercantile marine, than that which we published yesterday. Two Americans, the mates of the bark 'Anna,' an American vessel, were brought before the magistrates of the Isle of Wight on the charge of causing the deaths of six coloured men by a series of the most atrocious cruelties. We may say at once it was asserted that yellow fever had prevailed on board the ship, and that possibly the death of more than one of the men was due to this disease. But, if the evidence is to be believed, the deaths of at least two of them were directly due to the acts of the accused. The first part of the statement made by John Thomas, one of the surviving coloured men, relates to the murder of James Armstrong. Lane, the chief mate, gave an order to this unhappy creature. He did not attend to it as quickly as the mate wished, and Lane, taking up a mallet, struck him with it over the eye. The man 'jumped up, fell on the main deck with his head forward, and then leaned over the chain. I went to his assistance, put my hand on his head, and pulled it back, and I saw that his left eye was running out.' Armstrong was then sent half insensible 'down on the martingale under the bowsprit to clean the earring.' He was washed off the martingale and towed along in the water by the earring, which his arm was coiled round. As Abraham Rock, another coloured seaman, was about to haul him in, the chief mate said, 'Don't haul that nigger in; cut the earring, and let him go!' About two minutes after Armstrong let go his hold, and was lost. Another man, John Turtle, was dragged down by Hires, the second mate, who stamped on his head with his sea-boots. Turtle died, and the witness swears that he found the bone of the forehead broken in the centre. A youth named Johnson and a man named Frank also died after being ill-treated in the most frightful manner by the mates, and, though the deaths may not have been immediately the result of the beating and the choking, yet, supposing the negroes to have been in a weakly state from fever, there can be little doubt that such usage must have tended to produce fatal consequences. In all six coloured men perished, and their deatbs were all charged to the mates by the surviving seamen.

"As the offences were committed on board an American vessel and on the high seas, the American Minister in this country sent a protest against the jurisdiction of the Court, and nothing remained for the magistrates but to dismiss the charge. This protest was accompanied with a request that the magistrates would detain the defendants until the matter could be inquired into by the American Consul, so that they might be remitted to their own country for trial under the Extradition Treaty. The magistrates, however, considered that they could not hold them in custody until the formal requisition had been made, and so they were discharged, and will probably take care to put themselves out of the power of any English authority. In this the magistrates had no discretion; but we cannot think that they acted wisely or humanely in sending the witnesses back on board the American vessel, into the power of their persecutors, who may at any time set sail from Cowes and gratify their vengeance on the men who have endeavoured to bring them to justice. Whatever agreement may have been entered into by the seamen, the proceedings on board during the voyage are quite sufficient to justify them in refusing to fulfil it, and it is not the duty of an English bench of magistrates to force men to expose themselves to such treatment as caused the deaths of Armstrong and Turtle.

"We know not what view the American authorities take of such outrages as those we have detailed. It is quite possible that, should the two mates be sent to the United States under the Extradition Treaty, they will be acquitted, their victims being only 'niggers.' But these crimes for which the American merchant service has become justly infamous, demand the serious attention of a civilized people. Deeds of violence cannot be perpetrated with impunity without quickly demoralizing the community which suffers them. The barbarous practices which have sprung up within the last few years will, if not checked, rear up in every port of the Republic a set of desperadoes such as has not been seen in Europe since the days of buccaniers and pirates. In a few years an American merchantman will be a floating hell. Every boy who goes on board will learn the horrible lesson, and by the time he has strength to use marlingspikes and 'knuckle-dusters' he will be too bad for anything but the gallows. Nor let the Americans suppose that it is only 'niggers' who will be the victims of these savage instincts. The ruffian Moody, who was sentenced a few weeks since by an English Court to penal servitude for life, had tortured to death a brother American against whom he conceived a dislike, and there is ample evidence to show that in the madness of cruelty the petty despots of a merchantman are no respecters of persons. The authorities of this country have fully made up their minds on the subject. Every man who commits a murder on board a British ship, or on board any ship in British waters, will be prosecuted and hanged or otherwise punished. As a Christian people, as a people who believe in the sacredness of human life, we shall put the law in force, and protect the helpless seaman against those who so abuse the power they are intrusted with."

We have linked the prevalence of violence in the mercantile marine of America with those extraordinary scenes of violence which from time to time disfigure the social life of some of the most important cities of the States. For example, a little while ago we read a horrible scene which occurred in one of the hotels of New Orleans. Two gentlemen, who had had a previous disagreement, came accidentally into contact one day after dinner, whereupon both drew pistols from their breasts, and at once commenced to fire at each other. The account runs:—

"The firing having commenced, Harris retreated, and finally dodged into the door of the small bar and cigar-room, and shielding himself partly behind the glass door, looked out and fired from time to time. Peck, while Harris retreated, stepped out from the office and fired several shots—three of which took effect upon the person of Harris—and was in that position when he was fired at from the room. Exhausting his pistol, Peck drew his bowie-knife and advanced deliberately toward the door of the cigar shop, from behind which Harris had shot, and seemed to hesitate a moment whether to enter. The next moment Harris fired at the open doorway, the ball of his pistol entering the side or jamb of the door. After firing the last shot, Harris ran back just as Peck entered the door and got over the marble counter of the bar, and into a corner among the bottles. Peck following, sprang over after him, and grasping hold of him, inflicted upon his person four stab wounds with the bowie-knife. Harris was picked up and placed on the floor for a few moments, and then carried to his room near by, expiring almost the moment he was placed upon his bed. The excitement was very intense, and most of the crowd got out of the way at the first firing. Some got behind pillars, others ran into the passages leading to the dining-room and ladies' parlour, and not a few, thinking it too late to fly, made shields of the chairs. A group of gentlemen were standing conversing immediately in a line with the shot from Harris, which lodged in the wall a few feet above their heads. Harris received the following wounds:—One shot wound in the right shoulder, two stab wounds in the left shoulder, one stab wound in the left arm, one stab wound in the left side, between the fifth and sixth ribs, penetrating the lungs; one shot wound in the right side, between the seventh and eighth ribs, penetrating the liver. The two wounds last above-mentioned were the immediate cause of death. One shot wound in the breast, between the first and second ribs. Peck was arrested immediately after the murder."

Certain remarks of Dr. George Cook, of Brigham Hall, Canandaigua,

N.Y., in an admirable article on Mental Hygiene,[3] may, perhaps, serve to throw some light on the peculiar psychical phenomena indicated by the murderous instances which we have briefly recorded. After speaking strongly of the too prevalent weakening in the States of domestic ties between parents and children, and the unhappy effects which this exercises upon the moral tone of the latter, he makes the following observations :—

"We proceed to speak of the theory and practice of our boasted republican civilization, whereby the healthy rule in regard to the influence of parental and other example, upon the formation and development of mental and moral character, though recognised theoretically, is, in numberless instances, nullified in all its practical operations. In this connexion we would call especial attention to the importance of faith as an element of mental and moral health and power; faith in the good and true in man; faith in the goodness, truth, justice, and daily guidance of a Heavenly parent, to whom we owe reverence and obedience. The great minds that have arisen in the world's history; that have influenced and controlled the destinies of untold millions of their fellow-men; given form to their social, political, and religious institutions; contributed in the highest degree to the development of the arts and sciences, and to the progressive welfare of the human race, have derived no inconsiderable portion of their power and influence over other minds, from the faith which infused their own. If such a faith be an important element in strong and healthy character, it follows that a social life, which scatters widely the seeds of distrust and doubt, which develops precocious ideas of independence and freedom, instead of dependence and obedience, and allows the youthful mind to become familiar with evil in some of its most alluring forms before it is prepared to judge intelligently, cannot exert other than a vicious, unhealthy influence upon the physical, moral, and intellectual well-being of the present and future generations. That very many of our youth are exposed to such malign influences, at a period of their lives when they should be most carefully shielded from them by parental example and care, is but too plainly exemplified in the prominent traits of character peculiar to 'young America,' and in the steadily increasing stream of youthful depravity, crime, and disease. Later in life we may trace the evil through our whole social fabric, everywhere a prolific source of unhappiness, suffering, wrong-doing, and disease, both physical and mental.

"There are some, but they compose a small and scattered minority in many communities, who correctly appreciate the importance of a stable foundation, on which to rear the superstructure of physical, moral, and intellectual manhood. They are worthy of all honour for their steadfastness of purpose and adherence to the right, in the midst of the general laxity which so universally prevails."

Before terminating our Retrospect, we cannot avoid directing attention to a case which occurred early in the quarter, in the Westminster Police Court, and which is a singular illustration of popular information concerning lunacy.

A middle-aged man, who believed that he was Oliver Cromwell, occupied lodgings in Westminster. He was supposed to be under a sort of surveillance by the landlord, although it appeared that he had

[3] *American Journal of Insanity*, January, 1859.

almost uncontrolled command of his actions in going in and out of the house. It further appeared that the lunatic had certain warlike propensities, and that he was permitted, in the indulgence of these propensities, to arm himself with sundry murderous steel weapons, made and even ornamented to his own pattern. One morning he quietly entered the room where his landlord was seated, and, going behind him, felled him to the earth with a tremendous blow, given by a heavy steel bar, one of the warlike implements referred to. The landlord was much hurt, but fortunately for him the blow did not prove fatal. In due time the lunatic was arraigned before the magistrate for assault, and the instructive portion of the business was this ; the landlord asserted that until he was struck down he had never for a moment thought that the lunatic was dangerous. Notwithstanding that the lunatic had a fancy for offensive weapons, and expected every moment to be called upon to defend his rights or assail the rights of others, still it had not occurred to his keeper that he was dangerous !

This was an exceedingly narrow escape from another horrible butchery by a homicidal maniac at large, and it ought to teach the press, who are too apt to cavil with the alienist who is desirous of placing lunatics of this species under durance or strict watch, that it is only by sequestration or constant surveillance that the public can be protected from their dangerous tendencies.

